# Prevalence and associated factors of foot deformity among adult diabetic patients on follow-up at Debre Markos comprehensive specialized hospital, Northwest Ethiopia, 2022, cross-sectional study

**DOI:** 10.1186/s12902-023-01519-8

**Published:** 2023-11-30

**Authors:** Aderajew Agmass Adebabay, Amanuel Girma Worede, Bickes Wube Sume, Getachew Tilaye Mihiret, Rahel Asres Shimelash, Bahiru Tenaw Goshu

**Affiliations:** 1 Department of Biomedical science, School of Medicine, College of Medicine and Health Sciences, Debre Markos University, Debre Markos, Ethiopia; 2 Department of Human Anatomy, School of Medicine, College of Medicine and Health Sciences, University of Gondar, Gondar, Ethiopia; 3 Department of Midwifery, Medicine and College of Health Sciences, Debre Markos University, Debre Markos, Ethiopia; 4 Department of pediatric Nursing, Medicine and College of Health Sciences, Debre Markos University, Debre Markos, Ethiopia

**Keywords:** Diabetes Mellitus, Foot deformity, Prevalence, Associated factors, Ethiopia

## Abstract

**Introduction:**

Diabetes foot deformity is among the major causes of diabetic foot ulceration, resulting in lower limb amputation. However, the study on the distribution of foot deformity and its risk factor among diabetic patients in Ethiopia is limited. This study determined the overall prevalence and associated factors of foot deformity among adult diabetic patients on follow-up at Debre Markos Comprehensive Specialized Hospital, Northwest Ethiopia.

**Methods:**

Hospital-based cross-sectional study was conducted among 392 diabetic patients using a systematic random sampling technique at Debre Markos Comprehensive Specialized Hospital. Data were collected by pre-tested, semi-structured questionnaires and diabetic foot assessment format. Multivariable binary logistic regression was used to determine the association between dependent and independent variables. Adjusted odds ratios (AOR) with their 95% confidence interval (CI) were used to determine the strength of the association, and a variable with a p-value < 0.05 was statistically significant factors of diabetes foot deformity.

**Result:**

The overall prevalence of foot deformity was 33.4% [95% CI: 28.9–38.3]. In the final logistic regression analysis, rural residency [AOR = 2.64, 95% CI: 1.31, 5.31], poor glycemic control [AOR = 2.41; 95% CI: 1.34, 4.33], diabetes duration ≥ 10 years [AOR = 2.74; 95% CI: 1.50, 5.02], inadequate footwear [AOR = 2.11; 95% CI: 1.17, 3.82] and presence of peripheral neuropathy [AOR = 8.21; 95% CI: 4.54, 14.84] were statistically significant associated factors with diabetes foot deformity.

**Conclusion:**

The prevalence of foot deformity among adult diabetic patients was high. It is recommended to incorporate foot deformity screening in routine diabetic patient follow-ups especially for those with poor glycaemic control, rural residency, long diabetes duration, inadequate footwear, and diabetic peripheral neuropathy.

**Supplementary Information:**

The online version contains supplementary material available at 10.1186/s12902-023-01519-8.

## Introduction

The foot is a highly complex terminal structure of the lower extremity [1]. It comprises many bones, muscles, joints, ligaments, tendons, and neurovascular structures from each foot [2]. It provides support when standing and moving, and allows adjustment to the unevenness of the ground [1, 2]. Normal gait needs sensory input to adapt and modify motor patterns and muscle output to carry out the desired task [3]. Gait quality is closely associated with the overall state of health [4].

The normal foot anatomy may altered by diabetes mellitus (DM), which primarily damages the peripheral neurovasculatures [5]. Damage to the intrinsic foot muscles’ innervation results in an imbalance between flexion and extension of the affected foot. This produces anatomic foot deformity and abnormal foot prominence [6]. Changes in foot posture and architecture induced by diabetes will impact the normal biomechanics of walking and weight-bearing of the foot [7, 8]. The tendon and capsule of the diabetic patient undergo structural alterations, and the ligament, capsule, and tendons of diabetics have an erratic pattern [9] resulting in deformed foot.

Foot deformities, which are structural abnormalities of the foot, such as claw or hammertoe, callus, hallux valgus, pes cavus, pes planus, prominent metatarsal heads, Charcot foot, and amputation, are one of the biomechanical alterations common to the diabetic foot [7, 8]. Diabetic foot deformity is one of the most common predictors of diabetic foot ulceration, leading to lower limb amputation [10, 11]. It is also one of the main causes of disability and death in diabetic patients [12]. Diabetes causes severe and diffuse disease below the knee, and the lifetime risk of developing a diabetic foot ulcer is between 19% and 34% [13]. Diabetic foot disease eventually affects up to 50% of patients with both type 1 and type 2 diabetes [14].

According to World Health Organization (WHO) global reports in 2019, diabetes appears to intensely increase the risk of lower extremity amputation because of infected, non-healing foot ulcers [15]. In 2016, the Global Report on Diabetes showed that lower limb amputation rates were 10 to 20 times higher among people with diabetes than non-diabetics [13]. As the world is facing an increasing incidence of type 1 and type 2 DM, the International Diabetic Federation (IDF) chose to focus on the global burden of diabetic foot disease in 2005 [16]. According to IDF reports, a lower limb is amputated every 20 s due to diabetes [13].

In Ethiopia, ulcer of the foot is a major cause of disability, morbidity, and mortality among diabetic patients and about 15% develop foot ulcers in their lifetime [17]. The identified significant associated factors of diabetic foot ulcers (DFU) are the presence of foot deformity, increased BMI, advanced age, poor glycaemic control, and poor self-care practice [18, 19]. Diabetic-related foot deformity have been documented as a causal pathway for developing DFUs in patients that have developed diabetes related peripheral neuropathy (DPN) and impaired circulation at the periphery [20, 21].

A number of recognizable structural foot abnormalities are related to diabetic foot problems. It can happen alone or in groups, exposing people to diabetes-related foot complications. A diabetic foot assessment checklist is not used by medical practitioners in the health institution in the study area. The early detection of diabetic foot deformity is a crucial indicator of those who are more likely to develop foot ulcers [22]. Therefore, integrating foot deformity screening and its associated factors in routine diabetic foot programs will facilitate early detection and prevention of diabetic foot deformity. This is ultimately important to reduce the occurrence of diabetic foot ulcer.

There is limited evidence on the prevalence of foot deformity and its contributing factors among diabetic patients in Ethiopia. In order to better understand the prevalence and associated factors of foot deformity in adult diabetic patients, this study was conducted at Debre Markos Comprehensive Specialized Hospital in Northwest Ethiopia, 2022.

## Methods

### Study design, area and period

A hospital-based, cross-sectional study was implemented to assess the prevalence and its associated factors of foot deformity among adult diabetic patients at Debre Markos Comprehensive Specialized Hospital from June 1st to July 30th, 2022. This hospital is found in Debre Markos town, the capital city of East Gojjam Zone, 300 km away from Addis Ababa, Northwest Ethiopia. It provides health services to more than five million people and as a teaching service for Debre Markos University. In the hospital, twelve different specialized units provide outpatient services, including chronic follow up for diabetes mellitus, which serves around 4620 diabetic patients annually.

### Source population

All adult patients with diabetes mellitus who attended outpatient follow-up at Debre Markos Comprehensive Specialized Hospital (DMCSH) were our target populations.

### Study population

During the study period, all adult diabetic patients who came to DMCSH for outpatient follow-up were included.

### Sample size determination and sampling technique

Using a single population proportion formula, the required sample size was calculated under the following assumptions: the prevalence of overall diabetic foot deformity was 36.5% (p = 0.365) obtained from previous study conducted in South west Ethiopia [19], standard normal distribution value at 95% confidence level and 5% tolerable error (d = 0.05). Then, considering 10% for the non-response rate the final sample size was 392. The study participants were selected by systematic random sampling technique. We identified the average patient flow at the diabetic outpatient follow up based on the previous year’s data. Around 4620 diabetic patients per year had diabetic follow-up at DMCSH. The entire number of patients was divided into twelve months, yielding an average of 770 diabetic patients per two month. Then we followed the guidelines of the systematic random sampling approach to obtain the study unit. As the source population, we considered the previous two-month diabetic patient flow (N). Then it was divided by the total calculated sample size (n) to get the constant (K).

$$\text{K}=\frac{N}{n}=\frac{770}{392}=1.96\sim2$$

We used lottery method to select the first respondent in order to start the interview and clinical examination. Then, every two patients were recruited depending on their entrance order until the required sample (392).

### Eligibility criteria

#### Inclusion criteria

All patients aged 18 or older with the diagnosis of diabetes mellitus (type 1 or type 2) and at least six fasting blood glucose measurements in the past six months were included.

#### Exclusion criteria

Diabetic patients who had a congenital foot deformity or foot deformity caused by trauma, known rheumatoid arthritis, complicated current foot ulcers or were critically ill who were unable to give informed consent during data collection were excluded.

### Study variables

#### Dependent variable

Foot deformity.

#### Independent variables

**Socio-demographic** factors include age, sex, level of education, occupation, income, and residency.

**Behavioral factors** smoking, alcohol use, and physical activity.

**Clinical factors** type of DM, duration of DM, glycaemic control, footwear, body mass index (BMI), level of blood pressure, and other DM complications such as DPN and PVD.

### Operational definition

**Foot deformity** was defined as the existence of any of the following structural abnormalities on either or both feet, such as hammer/claw toe, hallux valgus, prominent metatarsal heads, pes cavus, Charcot foot, and amputation in diabetic patients [23, 24] (Table S1). Note:- a deformity was either present or absent without attempting to level severity.

**Congenital foot deformity** is deformity that develop at or before birth [25], **foot deformity caused by trauma**: deformities due to injury, accidents, or infection [26].

#### Past smoker

Someone who had previously smoked more than 100 cigarettes throughout their life. **Current smoker**: someone who has regularly smoked at least one cigarette per day for at least one month. **Non-smoker**: never smoked anywhere in his or her life [27].

#### Physically inactive

Participants who were well-functioning a moderate-intensity activity for less than 150 min, vigorous intensity for less than 75 min, or less than five days of any combined walking, moderate-intensity, or vigorous-intensity activities [28].

#### Alcohol users

A respondent who drank more than four standard units for males and 3 standard units for a female of alcohol per day [29].

#### Poor glycaemic control

Respondents who had average fasting blood glucose level greater than 130 mg/dl for six months [30–32].

#### Diabetic peripheral neuropathy

was diagnosed that a patient with a history version of Michigan neuropathy-screening instrument questionnaire scores > 7, or if the participants were lost feeling of vibration by tuning fork [33].

#### Peripheral vascular disease

was identified if the tibialis posterior pulse was absent, either alone or in conjunction with other lower extremity vascular signs or symptoms, or **An absent dorsalis pedis pulse** with at least one lower limb vascular symptoms/signs, such as claudication, rest pain, edema, leg numbness, and pale and mottled skin [34].

**High blood pressure** was defined as systolic blood pressure ≥ 140 mmHg and/or ≥ 90 mmHg on two occasions four hours apart, or known hypertensive on treatment.

**Inadequate footwear**: was characterized as having at least one of the following characteristics: bare feet, too tight or wide, high heels, poor quality leather, or soft insoles for diabetics [35].

### Data collection tool and procedures

Following ethical approval, data were collected using interviewer administered semi-structured questionnaires by trained professional nurse, a medical doctor and one senior orthopaedic surgeon. The questionnaire included socio-demographic data, behavioural factors, clinical factors and diabetic foot assessment checklist, which were adopted from WHO step wise surveillance of non-communicable diseases [36]. We also used digital sphygmomanometer, Standard weight scale, standard height scale for measurement of blood pressure, weight and height respectively. United State American (USA) manufactured 128 Hz tuning fork was used to diagnose diabetic peripheral neuropathy. Study participants were monitored to determine if they met the inclusion criteria. Socio-demographic, behavioural and clinical data, as well as measurements of blood pressure, weight and heights were collected by trained professional nurse according to WHO guidelines [37]. Body mass index was calculated by dividing each patient’s body weight by the square of their height in STATA 14. We defined **underweight** as less than 18.5 kg/m^2^, **normal range** as 18.5–24.5 kg/m^2^, **overweight** as 24.5 to 30 kg/m^2^, and **obese** as greater than 30 kg/m^2^ [24]. Secondary data were also used to collect type DM, duration of DM, previous fasting blood sugar and drug related data from patient chart.

Diabetic foot assessment checklists included Michigan neuropathy-screening instrument were done by medical doctor and finally foot deformity were ascertained by senior Orthopedic surgeon through careful inspection and palpation techniques. The data collectors used the patient’s Medical Registration Number (MRN) as a code and asked and verified whether the patient had been interviewed or not before data collection in order to avoid repeating patients with repeated visits.

### Data quality control

The questionnaires were adopted from WHO stepwise surveillance of non-communicable diseases [36] and applied in different similar studies.

It was prepared in English and then translated to the local language (Amharic) and then back to English to keep its consistency. Pre-test was done among 20 of sampled study participants at Finote Selam General hospital. Two days training was provided to data collectors about the purpose of the study, how to collect data, and examine foot deformity in diabetic patients. Every day, data collectors were closely supervised. Finally, the gathered data were double-checked to make sure they were accurate, clear, and consistent.

### Data process and analysis

The collected data were first entered, cleaned, and coded in epidata version 4.6 before being exported to STATA software version 14 for analysis. Descriptive statistics were used to present and summarize the data in the form of the median, interquartile range, frequency and percentages in tables and graphs with 95% confidence intervals for prevalence estimates.

A binary logistic regression model was used to identify factors associated with diabetic foot deformity among the study participants. A Collinearity diagnostic test as well as Hosmer and Lemeshow model goodness-of-fit tests were performed. Variables that showed an association with foot deformity in the bivariable analyses at *p* < 0.25 were entered into the multivariable logistic regression model. The association between independent variables and dependent variables was investigated using multivariable binary logistic regression analysis while controlling for other potential confounders. To assess the strength of the association, an adjusted odds ratio with a 95% confidence interval was calculated. In multivariable regression analysis, variables with a p-value less than 0.05 were considered statistically significant factors.

## Results

### Socio-demographic features of participants

There were 392 participants in this study. Female respondents made up more than half (53.83%) of the total. The median age of the respondents was 47 (Inter Quartile Range (IQR) = ± 19) ranging from 18 to 82 years. One hundred and six (27.04%) responders were between the ages of 50 and 59. A little over 54.34% of respondents resided in urban areas. Considering the participants’ present employment, 27.04% were farmers (Table 1).

**Table 1:** Socio-demographic features of study population (n = 392)

Variables	Category	Foot deformity		Total frequency (%)
		Yes	No	
Sex	Male	70	111	181 (46.17)
	Female	61	150	211 (53.83)
Age	18–29 years	7	44	51 (13.01)
	30–39 years	17	61	78 (19.90)
	40–49 years	33	68	101 (25.77)
	50–59 years	45	61	106 (27.04)
	>=60 years	29	27	56 (14.29)
Residency	Rural	78	101	179 (45.66)
	Urban	53	160	213 (54.34)
Educational status	Unable to read and write	36	40	76 (19.39)
	Informal education	30	30	60 (15.31)
	Completion of primary school	23	63	86 (21.94)
	Completion of secondary	30	79	109 (27.81)
	College/university	12	49	61 (15.56)
Occupation	Farmer	45	61	106 (27.04)
	Merchant	23	72	95 (24.23)
	Government employee	23	57	80 (20.41)
	NGO/private employee	15	33	48 (12.24)
	Housewife	16	25	41 (10.46)
	Others*	9	13	22 (5.61)
Average monthly income (ETB)	< 1500	10	19	29 (7.4)
	1500–2999	25	40	65 (16.58)
	3000–6000	67	130	197 (50.26)
	> 6000	29	72	101 (25.77)

ETB: Ethiopian Birr, NGO: Non-Governmental Organization, *Student, Daily labour, Retired

### Behavioral features of participants

The majority of those who responded (93.62%) did not smoke. In regard to alcohol consumption, approximately 31.38% of respondents used alcohol. Nearly two-thirds (66.33%) of respondents were physically active, which means they engaged in more than 150 min of moderate or 75 min of vigorous physical activity per week (Table 2**)**.

**Table 2:** Behavioural characteristics of study participants (n = 392)

Variables	Category	Foot deformity		Total Frequency (%)
		Yes	No	
Smoking	Non-smoker	121	246	367 (93.62)
	Current smoker	6	12	18 (4.59)
	Past smoker	4	3	7 (1.79)
Alcohol drinking	Yes	49	74	123 (31.38)
	No	82	187	269 (68.62)
Physical activity	Physically active	71	189	260 (66.33)
	Physically inactive	60	72	132 (33.67)

### Clinical features of participants

Among study participants, more than two-thirds (68.11%) were diagnosed with type 2 diabetes mellitus (T2DM). With a minimum of 1 year and a maximum of 21 years, the median diabetes duration was 8 (IQR = ± 8) years. About 235 (66.58%) of respondents had diabetes duration of less than 10 years. The median fasting blood glucose was 135 mg/dl (IQR = ± 22). Of the total respondents, 54.85% had poor glycaemic control. Regarding the body mass index of respondents, 43.37% and 13.27% were overweight and obese respectively. Around 46.68% of respondents were diagnosed as hypertensive. Many of the respondents (45.41%) had taken Insulin followed by 37.50% of Oral hypoglycaemic users. More than half (54.85%) of the participants had inadequate footwear practice. One hundred fifty-nine (40.56%) participants had DPN diagnosed using the composite MNSI symptom score and lost vibration perception at either of the great toe, medial malleoli, or lateral malleoli. Fifty-eight (14.8%) respondents had peripheral vascular diseases (Table 3).

**Table 3:** Clinical features of study respondents (n = 392)

Variables	Category	Foot deformity		Total Frequency (%)
		Yes	No	
Type of DM	TIDM	28	97	125 (31.89)
	T2DM	103	164	267 (68.11)
Diabetic duration	< 10 years	54	181	235 (66.58)
	≥ 10 years	77	80	157 (33.42)
Glycaemic control	Poor control	88	127	215 (54.85)
	Good control	43	134	177 (45.15)
BMI	Normal	44	117	161 (41.07)
	Underweight	1	8	9 (2.30)
	Overweight	62	108	170 (43.37)
	Obese	24	28	52 (13.27)
Hypertensive	Yes	55	154	183 (46.68)
	No	76	107	209 (53.32)
Current medication	Insulin	58	120	178(45.41)
	Oral hypoglycaemic	51	96	147 (37.50)
	Mixed(insulin and oral)	21	40	61 (15.51
	Prescribed diet only	1	5	6 (1.53)
Foot wear	Adequate	37	129	166 (42.35)
	Inadequate	94	132	226 (57.65)
DPN	Yes	97	62	159 (40.56)
	No	34	199	233 (59.44)
PVD	Yes	39	19	58 (14.80)
	No	92	242	334 (85.20)

Note: DPN: Diabetic Peripheral Neuropathy, PVD: Peripheral Vascular Disease

### Prevalence of foot deformity among study participants

The overall prevalence of foot deformity among adult diabetic patients who had a follow-up at DMCSH was 33.4% [95% Cl, 28.9–38.3]. The prevalence of foot deformity among type I and type II adult diabetic patients were found to be 22.4% and 38.6% respectively (Table 3). Most of the study respondents were having more than one deformity. The most frequent type of foot deformity reported in this study was hammer/claw toe (44.3%) followed by prominent metatarsal head (26.72%). the lowest prevalence was charcot foot, which was present in only two (1.53%) of the participant (Table 4).

**Table 4:** Types of foot deformities among study respondents

Foot deformity (n = 131)	Frequency	Percentage (%)
Hammertoe /claw toe	58	44.3
Hallux valgus	33	25.19
Pes cavus	16	12.21
Prominent metatarsal heads	35	26.72
Charcot’s foot	2	1.53
Amputation	7	5.34

### Factors associated with foot deformity

In the bivariable binary logistic regression model, variables having an association with foot deformity at a P-value of less than 0.25 were sex, age category, residence, educational status, alcohol drinking, physical activity, type of DM, duration of DM, glycaemic control, hypertension, Body Mass Index (BMI), footwear, DPN, and PVD. These were entered in a multivariable binary logistic regression model to determine statistically significant factors associated with foot deformity among study participants.

However, in multivariable binary logistic regression analysis, five variables such as: residency, glycaemic control, duration of diabetes, footwear, and DPN were associated with foot deformity at P-value < 0.05. According to the study findings, respondents who live in rural area were 2.64 times higher at risk of developing foot deformity compared to those residing in urban areas (AOR = 2.64, 95% CI: 1.31; 5.31). When compared to diabetic patients with good glycaemic control, those with poor glycaemic control had a 2.41-fold greater risk of foot deformity [AOR = 2.41; 95% CI: 1.34; 4.33].

Diabetic patients who had been diagnosed for ≥ 10 years had a 2.74 times higher risk of developing diabetic foot deformity than those who had been diagnosed for less than 10 years [AOR = 2.74, 95% CI: 1.50, 5.02)]. Patients with diabetes who wore inadequate footwear had a 2.11-fold higher risk of developing diabetic foot deformities than those who wore adequate footwear [AOR = 2.11; 95% CI: 1.16, 3.81]. Furthermore, those diabetic patients who had diabetic peripheral neuropathy were 8.21 times more likely to develop diabetic foot deformity as compared to those diabetic patients without neuropathy. [AOR = 8.21; 95% CI: 4.54,14.84] (Table 5).

**Table 5:** Bivariable and Multivariable binary logistics regression analysis of factors associated with foot deformity (n = 392)

Variables	Category	Foot deformity		COR (95%CI)	AOR(95%CI)	p-value
		Yes	No			
Sex	Female	61	150	I	I	
	Male	70	111	1.55(1.02, 2.36)	1.34(0.74, 2.42)	0.342
Age	18–29 years	7	44	I	I	
	30–39 years	17	61	1.75 (0.66, 4.58)	1.21(0.36, 4.10)	0.749
	40–49 years	33	68	3.05(1.24, 7.49)	1.90(0.54, 6.67)	0.314
	50–59 years	45	61	4.63(1.9, 11.24)	2.74(0.76, 9.85)	0.121
	≥ 60 years	29	27	6.72(2.59,17.25)	2.35(0.56, 9.89)	0.243
Residency	Urban	53	160	I	I	
	Rural	78	101	2.33 (1.52, 3.58)	2.63(1.31, 5.31)	0.007*
Education	College/ above	12	49	I	I	
	Secondary	30	79	1.55(0.70, 3.31)	2.07 (0.68, 6.29)	0.198
	Primary	23	63	1.4 (0.67, 3.28)	1.47(0.53, 4.31)	0.473
	Informal	30	30	4.08 (1.8, 9.17)	1.05(0.37, 2.98)	0.931
	Unable to read and write	36	40	3.67(1.69, 7.98)	1.52(0.59, 3.94)	0.390
Alcohol	No	82	187	I	I	
	Yes	49	74	1.51(0.96, 2.36)	1.45(0.75, 2.83)	0.271
Physical activity	Active	71	189	I	I	
	Inactive	60	72	2.2(1.4, 3.44)	1.72(0.92, 3.21)	0.088
Type of DM	T1DM	28	97	I	I	
	T2DM	103	164	2.18(1.3, 3.54)	1.85(0.72, 4.73)	0.199
Duration of DM	< 10 years	54	181	I	I	
	≥ 10 years	77	80	3.23(2.09, 4.99)	2.74(1.50, 5.02)	0.001*
Glycaemic control	Good Control	43	134	I	I	
	Poor control	88	127	2.16(1.39, 3.34)	2.41(1.34, 4.33)	0.003*
BMI	Normal	44	117	I	I	
	Underweight	1	8	0.32(0.39, 2.75)	0.38(0.35, 4.31)	0.442
	Overweight	62	108	1.52(0.90, 2.43)	1.02(0.51, 2.09)	0936
	Obese	24	28	2.27(1.19, 4.34)	2.28(0.84, 6.15)	0.103
HTN	No	55	154	I	I	
	Yes	76	107	1.98(1.29, 3.04)	0.81(0.40, 1.61)	0.541
Footwear	Adequate	37	129	I	I	
	Inadequate	94	132	2.48 (1.58, 3.89)	2.11(1.16, 3.81)	0.013*
DPN	No	34	199	I	I	
	Yes	97	62	9.16(5.6, 14.85)	8.21(4.54,14.84)	< 0.001*
PVD	No	92	242	I	I	
	Yes	39	19	5.39 (2.96, 9.82)	1.45(0.66, 3.18)	0.349

COR: Crude Odds Ratio, AOR: Adjusted Odds Ratio, CI: Confidence Interval, I: Reference

* Statistically significant in the multivariable binary logistics regression at p-value < 0.05

## Discussion

Since foot deformity can result in an area of abnormally high plantar foot pressures, it plays a crucial role in the development of diabetic foot ulcers [38]. Understanding foot deformity prevalence and its risk factor in diabetic patients is an essential step in the prevention of further diabetic foot complications. The variables that had significant association with foot deformity were rural residency, poor glycaemic control, long duration of diabetes, inadequate footwear, and presence DPN.

According to this study findings, 33.4% of adult diabetic patients who went to a follow-up appointment at a diabetes clinic had foot deformity. This finding is in line with the results of studies conducted in Mizan Tepi, Southwest Ethiopia (36.5%) [19] and Jordan 34% [23]. However, the findings of this study are lower than those of studies conducted in Kenya, Iraq, the United Kingdom, India, Spain, and China, which found an overall prevalence of foot deformity among diabetic patients were 46% [39], 46.7% [40], 44.5% [19], 40% [41], 60.2% [42] and 42% [43] respectively. This difference could be due to variations in the study populations, study design, and assessment methods. For example, studies in India and Spain included only type DM with a smaller sample size, whereas we recruited both type 1 DM and type 2 DM, and respondents in China were older (mean age = 59.77 ± 11.83 years). Similarly, a study conducted in Philadelphia on 1000 diabetic individuals older than 65 years was also higher than the current study, which reported the prevalence of one or more foot deformities was 64.2% [44]. These variations might be due to the differences in the assessment method, which used X-ray images, or the age of the participants.

So far, the current study finding is higher when compared to a studies investigated in Egypt, Cameron, and northern Canada which stated the prevalence of foot deformity among diabetic respondents was 18.36%, 17.3%, and 23% respectively [45–47]. The possible justification for this inconsistency could be due to lifestyles and socio-economic variations among the study participants. The finding showed that diabetic patients who lived in rural areas had a higher chance of developing foot deformity than urban dwellers. The reason for this might be that patients who live in rural areas lack knowledge about self-care techniques. In addition, the majority of diabetic patients from rural Ethiopian areas work as farmers and walk barefoot on a regular activities, which leads to incorrect foot mechanics. Diabetes also causes the muscles in the feet to atrophy, which increases the risk of developing foot deformities in the patient. In contrast to this finding, study conducted in Iraq [40] residency was not an independent factor for foot deformity. This disparity might be attributed to the socio-demographic difference among study participants.

The current study demonstrates that poor glycemic control is significant risk factor for foot deformity. This is supported by a study conducted in Jordan, which revealed that poor glycaemic management increased the occurrence of Charcot foot deformity by six times as compared to those with good control of diabetes [48]. Poor glycaemic control may expose to persistent hyperglycaemic state that influences all body systems including muscles and joints of the foot. Structural changes occur within the tendon and capsule of the diabetic patient due to prolonged hyperglycaemic state that makes joint stiffness and immobility.

According to the results of this study, diabetic patients with a longer diabetes history were independently linked to the development of foot deformity. Studies from different fields also came to the same conclusion, supporting this fact [40, 48, 49]. The possible cause might be due to people with diabetes mellitus subjected to hyperglycaemic states for a longer period, and this cumulative glycaemic burden can have harmful effects on various parts of the body, including the skin and feet. Long-term hyperglycemia results in an interaction between collagen and glucose that produces Advanced Glycation End products (AGE) [50]. The collagen in the Achilles tendon, capsules, and ligaments of the foot becomes strong and rigid as a result of the build-up of this AGE, making the foot rigid and unyielding.

Since foot deformity is a long-term complication of diabetes mellitus that occurs mainly after the development of neuropathy and reduced circulation to the periphery, patients having a longer duration of diabetes had a high chance to develop foot deformity. Also, this study indicates that the use of inadequate shoes is the significant associated factor for the development of diabetic foot deformity which was in line with a study carried out in Jordan [48] and Iraq [51]. This could be caused by uneven pressure on the foot joints, which could disrupt normal foot mechanics, cause foot muscle activity to be disturbed, raise the risk of developing diabetic foot deformities, and increase the likelihood of foot injuries leading to ulcerations and amputations. In addition, inadequate footwear does not support and does not distribute the weight bearing evenly in the foot.

Peripheral diabetic neuropathy was a highly significant predictor of diabetic foot deformity in the current study. This result is in line with other studies [11, 48, 49, 52]. This might be due to hyperglycemia that affects the nerves and microvasculature of the foot and the integrity of foot arches. Since muscles, ligaments, and connective tissue protect the integrity of the foot’s arch [53], when motor neuropathy strikes, the muscles deteriorate and atrophy [6]. Due to the absence of the usual balance between the toe flexors and extensor muscles, a person may acquire foot deformities [53]. Similar to this, motor neuropathy typically exhibits structural changes to the dynamic anatomy of the foot and joints, leading to the wasting and weakness of small intrinsic muscles.

### Study limitations

Our study had some limitations despite filling a gap in the Ethiopian literature.

The assessment of foot deformity depend on the clinical examination by inspection and palpation method only, not supported by imaging methods that may show the degree and severity of foot deformity due to a shortage of budget and conducted in single setting may not be enough representative. Since the study is cross-sectional design, it does not show the cause and effect relation. There may also recall bias concerning the associated factors, such as tobacco smoking, alcohol use, or exercise frequency.

## Conclusion and recommendations

Generally, the findings of this study showed that the prevalence of foot deformity among adult diabetic patients was high. The variables that showed significant associations with diabetic foot deformity in the multivariable logistic regression were rural residency, poor glycaemic control, duration of diabetes, inadequate footwear, and peripheral neuropathy.

It is strongly recommended for clinicians to integrate foot deformity screening in routine diabetic management. Moreover, further researchers suggested to conduct by strong study design like a prospective cohort study in the multi-centre setting, to determine the cause and effect relationships, and provide best managements.

### Electronic supplementary material

Below is the link to the electronic supplementary material.

Supplementary Material 1

## Data Availability

The datasets used and/or analysed during the current study available from the corresponding author on reasonable request.
