# Supplementary material for: Prevalence and associated factors of foot deformity among adult diabetic patients on follow-up at Debre Markos comprehensive specialized hospital, Northwest Ethiopia, 2022, cross-sectional study
Source: BMC Endocr Disord. 2023 Nov 30;23:265. doi: 10.1186/s12902-023-01519-8 (PMC10687907; doi:10.1186/s12902-023-01519-8)
Supplement: Supplementary file 1 — Supplementary Material 1 [file 12902_2023_1519_MOESM1_ESM.docx]

**Table S1:** Types and definitions of foot deformities

| Foot deformity | Definition |
| --- | --- |
| Hallux valgus | Lateral deviation of the great toe at the metatarsophalangeal joint and pronation associated with bone prominence on the inner edge of the metatarsal (bunion) (1). |
| Hammer toes | Extension contracture at the metatarsophalangeal (MTP) and distal interphalangeal (DIP) joint with flexion contracture at the proximal interphalangeal (PIP) joint (2). |
| Claw toes | Hyperextension of the MTP with flexion of the PIP and distal interphalangeal (DIP) joint (3). |
| Pes Cavus | An excessively  high medial  longitudinal  arch, which extends between the first metatarsal head and the calcaneus approaching a right angle (4). |
| Charcot's foot | Presented with a low level of sensation, swelling, and foot associated with midfoot collapse or non-infectious destruction of bone and joint including loss of foot arches (5). |
| Prominent metatarsal heads | were defined as any inspected or palpable plantar prominences of the metatarsal heads of the foot (6). |
| Amputation | It was reported as any resection of segments of the lower extremity (7). |

**References**

1. Ray JJ, Friedmann AJ, Hanselman AE, Vaida J, Dayton PD, Hatch DJ, et al. Hallux Valgus. Foot & ankle orthopaedics. 2019;4(2):2473011419838500.

2. Mansour AA, Imran HJ, editors. Foot abnormalities in diabetics: Prevalence & predictors in Basrah, Iraq2006.

3. Walters DP, Gatling W, Hill RD, Mullee MA. The prevalence of foot deformity in diabetic subjects: A population study in an English community. Practical Diabetes International. 1993;10:106-8.

4. Ledoux WR, Shofer JB, Ahroni JH, Smith DG, Sangeorzan BJ, Boyko EJ. Biomechanical differences among pes cavus, neutrally aligned, and pes planus feet in subjects with diabetes. Foot & ankle international. 2003;24(11):845-50.

5. Sella EJ, Barrette C. Staging of Charcot neuroarthropathy along the medial column of the foot in the diabetic patient. The Journal of foot and ankle surgery : official publication of the American College of Foot and Ankle Surgeons. 1999;38(1):34-40.

6. Ababneh A, Bakri FG, Khader Y, Lazzarini P, Ajlouni K. Prevalence and associates of foot deformities among patients with diabetes in Jordan. Current Diabetes Reviews. 2020;16(5):471-82.

7. Ledoux WR, Shofer JB, Smith DG, Sullivan K, Hayes SG, Assal M, et al. Relationship between foot type, foot deformity, and ulcer occurrence in the high-risk diabetic foot. Journal of rehabilitation research and development. 2005;42(5):665-72.
